# Trustworthiness for an Ultra-Wideband Localization Service

**DOI:** 10.3390/s24165268

**Published:** 2024-08-14

**Authors:** Philipp Peterseil, Bernhard Etzlinger, Jan Horáček, Roya Khanzadeh, Andreas Springer

**Affiliations:** 1 Institute for Communications Engineering and RF-Systems, Johannes Kepler University Linz, 4040 Linz, Austria; philipp.peterseil@jku.at (P.P.); bernhard.etzlinger@jku.at (B.E.); roya.khanzadeh@jku.at (R.K.); 2 Institute of Networks and Security, Johannes Kepler University Linz, 4040 Linz, Austria; jan.horacek@jku.at; 3 LIT Secure and Correct Systems Lab, 4040 Linz, Austria; 4 JKU SAL IWS Lab, 4040 Linz, Austria

**Keywords:** trustworthiness, ultra-wideband, indoor localization

## Abstract

Trustworthiness assessment is an essential step to assure that interdependent systems perform critical functions as anticipated, even under adverse conditions. In this paper, a holistic trustworthiness assessment framework for ultra-wideband self-localization is proposed, including the attributes of reliability, security, privacy, and resilience. Our goal is to provide guidance for evaluating a system’s trustworthiness based on objective evidence, i.e., so-called trustworthiness indicators. These indicators are carefully selected through the threat analysis of the particular system under evaluation. Our approach guarantees that the resulting trustworthiness indicators correspond to chosen real-world threats. Moreover, experimental evaluations are conducted to demonstrate the effectiveness of the proposed method. While the framework is tailored for this specific use case, the process itself serves as a versatile template, which can be used in other applications in the domains of the Internet of Things or cyber–physical systems.

## 1. Introduction

Whenever systems interact with each other and with the physical world, preserving integrity to perform mission-critical tasks is essential. Therefore, in computer security, the concept of trust ensures that each component of software and hardware can be relied upon [1]. The notion of trust was adopted by the United States National Institute of Standards and Technology, which defined trustworthiness as one of nine essential aspects of cyber–physical systems [2]. The term has also been adopted by the Internet of Things (IoT) community [3], and has even been leveraged as a key value indicator for the International Mobile Telecommunications 2030 vision for the future 6G communication standard. While these developments highlight the importance of trustworthiness, it remains a vague term in the literature. Beyond a unified understanding of trustworthiness, practical frameworks that link high-level definitions to concrete realizations are lacking.

Ultra-wideband (UWB) localization services provide accurate positioning. The high bandwidth of typically 500 MHz, allows for precise distance measurements based on the time-of-flight of the radio signals. This technology is particularly advantageous for indoor environments where traditional GPS is ineffective. UWB localization offers centimeter-level accuracy, making it suitable for applications such as asset tracking and indoor navigation.

To close the gap in trustworthiness assessment, a systematic method is proposed for the IoT use case of UWB *self*-localization, i.e., specifically for a single node estimating its position relative to multiple anchors. This paper uses the following definitions:

**Trustworthiness** “Trustworthiness is the demonstrable likelihood that the system performs according to designed behavior under any set of conditions as evidenced by characteristics including, but not limited to, safety, security, privacy, reliability and resilience” [2].

**Trustworthiness metric** A trustworthiness metric is considered to be any measurement instance that describes the trustworthiness level of system operation.

**Trustworthiness indicator** Trustworthiness indicators map trustworthiness metrics to a likelihood interval in the range [0, 1], where 0 represents the lowest level of trustworthiness and 1 represents the highest level.

**Trustworthiness index** “A trust[worthiness] index is a composite and relative value that combines multiple trust[worthiness] indicators” [4].

**Threat** “Threat against a system refers to anything that can or may bring harmful effects to the state of the system and lead to improper service states” [5].

Self-localization is an essential service in IoT systems, creating dependencies to other components, services or entities [6]. Because UWB is used as the main technology for indoor localization [7], it is a perfect candidate for trustworthiness assessment. Our approach is threat-driven. First, threats to the system are identified and mapped to the attributes of trustworthiness. This correspondence may be used to ensure that no aspect of trustworthiness is missed in the evaluation. From these threats, measurable quantities are identified that indicate the presence of a threat. Using this approach, *meaningful* metrics are obtained (i.e., they correspond to realistic threats). Then, metrics are mapped to trustworthiness indicators in the value range from 0 to 1, with values below 0.5 being considered not trustworthy. The indicators are then combined in trustworthiness indices that represent the attributes of trustworthiness. By following this process, the contributions of this work are:

1. A general framework for trustworthiness assessment that can be adapted to various IoT applications using the presented assessment as a blueprint.
2. A threat-driven metric selection and indicator computation to identify meaningful system measures.
3. Experimental evaluation is conducted to provide insights into the strengths and weaknesses of the UWB self-localization service concerning trustworthiness, demonstrating that trustworthiness can improve the overall system performance.

Note that the proposed approach focuses on the novelty of the methodology, and does not claim completeness in terms of trustworthiness assessment.

### 1.1. Defining Trustworthiness

In this subsection, further key terms used throughout the article are introduced based on the previously provided definitions. As even the basic definitions vary in the literature, the focus is on capturing the essential characteristics of each definition.

**Figure 1:**
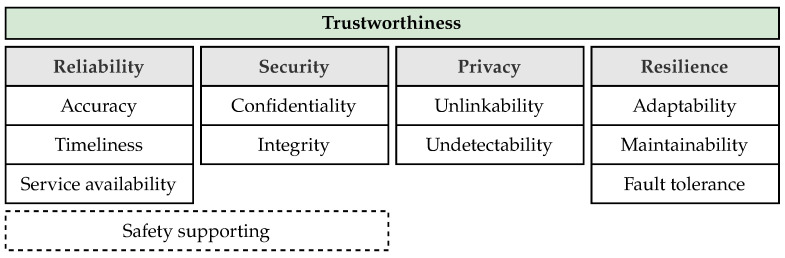
Taxonomy—attributes and sub-attributes of trustworthiness.

Trustworthiness is divided into five main attributes (sometimes called pillars or characteristics) [2,3]: safety, security, reliability, privacy, and resilience. In Figure 1, a graphical summary of these attributes is shown. The main focus of safety is to mitigate damage and harm to humans, objects, and the environment in which the system operates. Within the scope of this work (i.e., localization), safety is not considered a standalone attribute but is rather supported by reliability and security.

Traditionally, the primary goals of security are to protect the *confidentiality* (i.e., prevent unauthorized access), *integrity* (i.e., prevent unauthorized alterations), and *availability* (i.e., provide uninterrupted access to authorized subjects) of a system. For the purposes of this work, (service) availability is considered to fit better under the attribute of reliability, as the primary emphasis in security is on preventing malicious activities rather than merely ensuring operational functionality. Reliability is the ability of a system to provide a service under normal conditions, whereas resilience is the ability to adapt and recover from a state when a system is disrupted (e.g., by an attack). The related sub-attributes of reliability have the following meanings: *accuracy* is a measure of the deviation of a measurement from the true value, while *timeliness* refers to the ability to deliver results within the required time frame. The sub-attributes of resilience are *adaptability* (i.e., ability to adjust to new conditions), *maintainability* (i.e., the ease with which a system can be maintained and restored), and *fault tolerance* (i.e., the capability to continue operating properly in the event of the failure).

Privacy refers to the right of an individual to control access to and confidentiality of their personally identifiable information. In this setting, the focus is on protecting users’ location information and ranging data from unauthorized access or disclosure. *Unlinkability* is a property ensuring that different transactions cannot be associated with a specific user. *Undetectability* is an ability ensuring that a user’s presence cannot be identified.

Note that these attributes may overlap. For instance, reliability and resilience are sometimes considered as subsets of availability. However, the focus of these attributes is different. Availability generally focuses on maximizing uptime, while reliability deals with the probability of failures, and resilience emphasizes speed of recovery.

### 1.2. Related Work

The concept of trustworthiness has been studied across various communication domains, and substantial growth in relevance has recently been seen within the IoT and wireless sensor network research communities. To provide guidelines for developing trusted communication infrastructure and services, the Telecommunication Standardization Sector of the International Telecommunication Union (ITU-T) has published a recommendation that discusses the concepts, provision, and evaluation processes of trustworthiness [4]. Although this report introduces several trustworthiness attributes, it lacks clarity on how these attributes contribute to the overall assessment process. A more comprehensive set of attributes contributing to system trustworthiness is proposed in [5]. While key metrics for trustworthiness evaluation are discussed at the system level, the report does not offer quantitative approaches for assessing systems in operation. Focusing on industrial IoT applications, Ref. [8] identifies five main trustworthiness attributes, namely, reliability, security, privacy, resilience, and safety. The authors propose a generic framework for trustworthiness assessment based on these characteristics. However, they do not provide practical methods for implementing the assessment in specific industrial IoT applications or consider their unique requirements and limitations.

The notion of what constitutes a trustworthy system and how to assess the trustworthiness status of a system and its services highly depends on the specific application and the services that the system offers. In the realm of localization applications, a few studies in the literature have addressed trustworthiness evaluation from various perspectives. Considering only the reliability aspect of trustworthiness, Ref. [9] proposed an algorithm that integrates a trustworthiness index to evaluate the reliability of the information reported by nodes, thereby mitigating the impact of faulty nodes on localization accuracy. Ref. [10] presented a blockchain-based trustworthiness evaluation and management model for wireless sensor networks. The authors defined trustworthiness metrics, e.g., honesty and intimacy, which can be computed based on measurements from high network layers, e.g., the number of successful and unsuccessful interactions and the time of interaction. These metrics were used to evaluate the trustworthiness of anchors, which the nodes rely on for localization. While evaluating anchors’ trustworthiness is crucial, it is equally important to use meaningful metrics to assess the trustworthiness of all other entities in the network, including the nodes themselves, as well as the overall trustworthiness of the whole system. In [11], the authors presented a simulation framework focused on assessing the resilience of indoor ultrasound localization systems. However, their approach relies on metrics derived from the localization error, which requires ground truth information on the actual location, an impractical requirement in real-world implementations. A trustworthiness evaluation scheme for UWB communications was introduced in [12]. This scheme evaluates reliability and security using machine learning (ML) techniques; however, its reliance on only one metric, i.e., the channel impulse response, limits its ability to provide a holistic assessment of system trustworthiness.

In conclusion, existing studies such as [4,5,8] lack comprehensive quantitative assessment methods and practical implementation guidelines. They often focus narrowly on single aspects, such as reliability in [9], or assess resilience with impractical requirements, such as ground truth localization, as in [11]. Other studies, such as [10,12], perform trustworthiness assessment only on anchors and are based on limited metrics from higher network layers, which may not be readily available or cover all trustworthiness attributes. Additionally, approaches such as [12] assess reliability and security based on only one metric, for example, the channel impulse response, which fails to provide a holistic evaluation. Building upon these limitations in the current literature, we propose a structured, practical, and general trustworthiness assessment framework. This framework can evaluate the state of all involved entities in the network, including the anchors, nodes, and the overall system, from the aspects of reliability, security, resilience, privacy and safety. It can also be easily adapted to new use cases and applications. In addition, the proposed method utilizes a handful of metrics covering these aspects of trustworthiness and is driven by measurement data from lower network layers, thereby enhancing the generality and agility of the scheme.

### 1.3. Methodology

**Figure 2:**
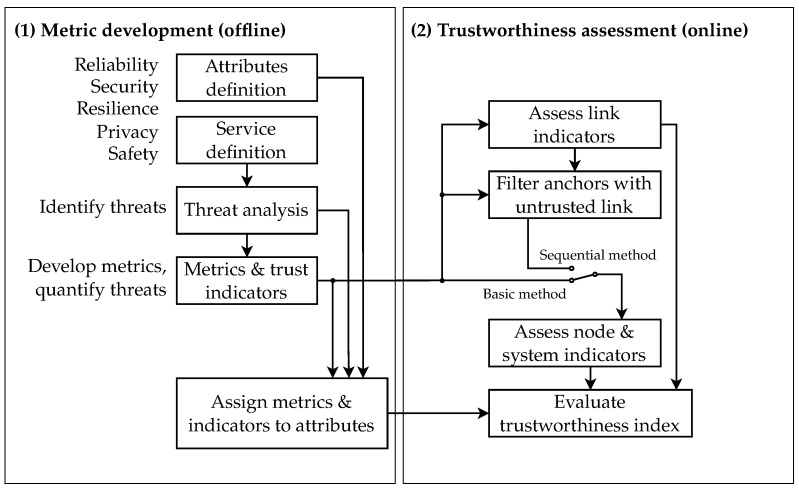
Methodology—the development of metrics (**left**) is carried out once during the offline phase, while trustworthiness assessment (**right**) is used during the online phase.

This study presents a methodology aimed at developing an application-centric trustworthiness assessment framework tailored specifically for UWB localization services. Figure 2 illustrates the workflow of the proposed methodology. By running through this workflow, trustworthiness can also be assessed for other IoT use cases, with the presented one serving as a blueprint. The proposed methodology consists of two main phases: metric development (Figure 2, left) and trustworthiness assessment (Figure 2, right). The final output is the trustworthiness index of the system.

The metric development phase links the general concept of trustworthiness to measurable quantities in the specific application. It consists of the following steps:

(i) Trustworthiness attributes: Trustworthiness is defined as a holistic measure that signals whether the system is working as intended. Hence, it first requires an understanding of which aspects of the system have to be observed. As proposed in literature [2,3], the currently considered system attributes include reliability, security, privacy, resilience, and safety (c.f. Section 1.1).
(ii) Service definition: To match the trustworthiness attributes to UWB self-localization, a clear understanding of the operational principle is required. This step establishes a clear and comprehensive understanding of the system under evaluation, laying the groundwork for the subsequent step of threat analysis.
(iii) Threat analysis: The threat analysis is a critical step aimed at identifying potential vulnerabilities of the defined service. It leverages critical parameters of the system at hand. The most challenging aspect is to address the entire set of identified trustworthiness attributes.
(iv) Metrics and trust indicators: While there are many possible measurable service parameters (referred to as metrics), this step identifies those that are relevant to detecting the likelihood of a threat. To make relevant metrics comparable, they have to be further mapped to trustworthiness indicators with a value range in the interval [0,1] where 0 and 1, respectively, indicate not trustworthy and trustworthy.
(v) Assign metrics and indicators to attributes: In order to obtain a measure for each trustworthiness attribute, the identified metrics and indicators first have to be assigned to the corresponding trustworthiness attributes. The indicators are then combined to indices that provide a high-level trustworthiness assessment.

Note that a coherent mapping between quantitative evaluation metrics and qualitative trust attributes can be established by defining potential threats and risks in UWB localization systems. This mapping ensures that the framework remains tightly aligned with the ultimate goal of trustworthiness assessment in UWB localization services and is achieved in the final step of the metric development phase (’Assign metrics and indicators to attributes’). Depending on which entity in the localization service is measured, the trustworthiness indicators are categorized as node (referring to the state of the node), link (referring to the state of the link between the node and each anchor), or system (referring to the state of whole localization system) indicators. Section 3, Section 4, and Section 5 respectively describe threat analysis, metric derivation, and assignment in detail.

The second phase of the proposed method is trustworthiness assessment, where the system’s trustworthiness is evaluated online. This evaluation involves monitoring the defined indicators and combining them to obtain a unified trustworthiness index for each attribute as well as an overall trustworthiness index for the entire system over time. Trustworthiness is assessed based on link, node, and system indicators. Two methods are proposed, called the basic and sequential methods. In the basic method, trustworthiness evaluation is conducted based on all metrics and their corresponding indicators, which are monitored in the system. In contrast, the sequential method involves filtering out anchors with untrusted links and then updating the node, link, and system indicators. This approach eventually enhances the robustness of the localization service, as shown in the results. The results reveal that considering trustworthiness in this way not only provides valuable insights into the system’s status but can also enhance its overall performance. In terms of scalability and deployment, trustworthiness is assessed locally on a node in a decentralized manner. The complexity does, therefore, not increase with the number of nodes present in the network but linearly with the number of available anchors.

## 2. Service Definition

A 2D self-localization service of one battery-powered node in an environment with multiple cable-powered anchors is considered. The service relies on UWB range measurements and subsequently processes the distance estimates (ranges) to obtain a location estimate. Finally, the location estimate can serve an application as a functional basis.

### 2.1. Range-Based Self-Localization

**Figure 3:**
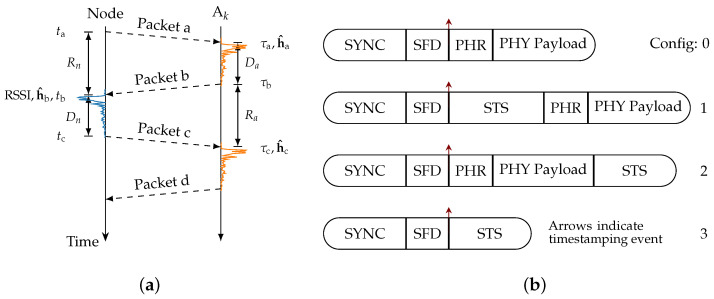
(**a**) Double-sided two-way ranging message exchange using four packets with channel measurements; the superscripts are omitted for simplicity, e.g., $t_{a}$ is used instead of $t_{a}^{\left(A\right)}$. (**b**) UWB packet configuration possibilities and the position of timestamping within the packet according to the IEEE 802.15.4z-2020 [16]. specification.

The node performs ranging with a subset $A_{\mathrm{eval}}=\left\{A_{1},\ldots ,A_{K}\right\}\subseteq A$ of all existing anchors A. The subset is selected either through the communication range of the node or through another criterion introduced later in Section 4.4. For ranging, three packets have to be exchanged; the measured time intervals are then converted into a range estimate. This packet exchange is referred to as *double-sided two-way ranging* [13]. In Figure 3a, the packet exchange cycle between node and anchor A∈A with the measured timestamps at the node $t_{a}^{\left(A\right)},t_{b}^{\left(A\right)},t_{c}^{\left(A\right)}$ and the anchor $\tau_{a}^{\left(A\right)},\tau_{b}^{\left(A\right)},\tau_{c}^{\left(A\right)}$, and channel impulse responses ${\hat{h}}_{a}^{\left(A\right)},{\hat{h}}_{b}^{\left(A\right)},{\hat{h}}_{c}^{\left(A\right)}$ is illustrated. Based on the round trip intervals, $R_{a}^{\left(A\right)}=\tau_{c}^{\left(A\right)}-\tau_{b}^{\left(A\right)}$ and $R_{n}^{\left(A\right)}=t_{b}^{\left(A\right)}-t_{a}^{\left(A\right)}$, and the response delays, $D_{a}^{\left(A\right)}=\tau_{b}^{\left(A\right)}-\tau_{a}^{\left(A\right)}$ and $D_{n}^{\left(A\right)}=t_{c}^{\left(A\right)}-t_{b}^{\left(A\right)}$, the ranges are computed according to [13] by

(1) $${\hat{r}}^{\left(A\right)}=c\;\frac{R_{a}^{\left(A\right)}R_{n}^{\left(A\right)}-D_{a}^{\left(A\right)}D_{n}^{\left(A\right)}}{R_{a}^{\left(A\right)}+D_{a}^{\left(A\right)}+R_{n}^{\left(A\right)}+D_{n}^{\left(A\right)}}\;,$$

where *c* is the speed of light. Processing time-of-flight according to (1) is known as *asymmetric* double-sided two-way ranging. The method provides implicit synchronization of node and anchor and does not impose constraints on the response delays $D_{n}^{\left(A\right)}$ and $D_{a}^{\left(A\right)}$. Detailed derivation can be found in the reference. The anchor A∈A is located at known position $x^{\left(A\right)}\in R^{2}$. The ranges are collected in $\hat{r}$ and the anchor positions in X, respectively:

$$\begin{array}{cc} \hat{r} & ={\left[{\hat{r}}^{\left(A_{1}\right)},\ldots ,{\hat{r}}^{\left(A_{K}\right)}\right]}^{⊤}\in R^{K}\;,\;\;\;\;\;X=\left[x^{\left(A_{1}\right)}\;┆\;\cdots \;┆\;x^{\left(A_{K}\right)}\right]\in R^{2\times K}\;. \end{array}$$

Anchor *A* uses the payload of packet b to share $\tau_{a}$, ${\hat{h}}_{a}$ and $\tau_{b}$ with the node. To provide it with $\tau_{c}$ and ${\hat{h}}_{c}$, a fourth packet is used.

For each localization sequence, the node first carries out ranging with all available anchors. Then, the node estimates its position x by computing

(2) $$\hat{x}=f_{\mathrm{loc}}\left(\hat{r},X\right)\;,$$

where the localization function $f_{\mathrm{loc}}$ can be chosen according to the system requirements. Here, a simple least-squares localization [14] is considered. The channel impulse responses and other channel-related features (e.g., the received signal strength indicator (RSSI)) are simultaneously recorded at the transceiver and frequently used for non line-of-sight detection [15].

### 2.2. UWB Packet Structure

According to the 802.15.4-2011 standard [7], the exchanged packets consist of four fields: the SYNC and SFD which together form the synchronization header (SHR), the physical layer header (PHR), and the PHY payload field for data transmission (see Figure 3b, configuration 0). Within the packet, a timestamping event is defined at the end of the SHR. It serves as a reference point for measuring the time intervals needed for (1). The exchanged physical layer (PHY) packets have one of the logical structures shown in Figure 3b, selected through node configuration. Configurations 0, 1, and 2 enable payload data to be appended, while configuration 3 serves purely for time measurements. Moreover, configurations 1, 2, and 3 include the scrambled timestamp sequence (STS) field, which adds an additional security mechanism. The use of the STS requires common knowledge of the keys and cryptographic scheme between transmitter and receiver. The location of the STS may vary depending on the configuration (see Figure 3b, configurations 1–3).

### 2.3. Receive Time Estimation for Ranging

Time measurements used for ranging refer to the moment the timestamping event occurs, i.e., when the end of the SHR appears at the antenna. The timestamp at packet reception is based on a leading-edge detection algorithm, denoted by $f_{\mathrm{LDE}}$, i.e.,

$$\mathrm{RX}\_\mathrm{STAMP}=f_{\mathrm{LDE}}(\mathrm{channel},\mathrm{SHR})\;.$$

While currently no UWB transceiver manufacturer discloses information about its leading-edge detection implementation, it is understood that these algorithms rely either on the received SHR or on the STS waveform, and that the accuracy is influenced by the propagation channel.

Implementations using the SHR sequences for timestamping are vulnerable to distance spoofing attacks. This can be prevented by using the STS sequence for timestamping, which is derived from a cryptographic function depending on a key only known to legitimate devices. However, Ref. [17] have demonstrated that injection of a random signal with high power at the same time as the legitimate transmitter sends the sequence can cause distance reductions in certain cases, as the sequence seems not to be evaluated bit-wise but only through correlation of the received signal with a template of the sequence.

### 2.4. Protocol Stack for Data Exchange

Communication is encoded in medium access control (MAC) frames as defined by IEEE802.15.4. The MAC layer offers two addressing modes, namely, extended unique IDs and short IDs, which are dynamically assigned upon association with a private area network. The standard also specifies an authenticated encryption with associated data method for MAC layer security, providing payload data confidentiality, authenticity, and replay protection. Additionally, an MAC frame can request acknowledgment from the receiver to confirm the correct packet reception; if not received, this triggers a retransmission.

### 2.5. Known Exploits for UWB Ranging

As UWB ranging is used in security-relevant applications, it is a target for attacks. Examples include distance reduction attacks for keyless entry systems. Attacks can occur on the node level (e.g., targeted battery drain), on the link level (e.g., early detect/late commit attacks), or on the system level (e.g., anchor node impersonation). Link-level attacks were specifically studied in [18,19,20].

## 3. Threat Analysis

In this section, some major threats to the UWB systems described in Section 2 are classified. Primarily, negative events that can disrupt one or more basic trustworthiness attributes of such systems are discussed. These threats can be divided into two main categories: *native threats* to self-localization (i.e., any system that uses UWB localization is subject to these threats), and *application-specific* ones (i.e., these threats depend on the broader context of a given application). The main focus of this paper is on native threats.

Furthermore, threats specific to nodes (i.e., the node itself), links (i.e., communication between a node and an anchor), or the system (i.e., localization service) are distinguished. Detailed overviews are provided in Table 1 for node threats, Table 2 for link threats, and Table 3 for system threats. Node threats (e.g., overheating) may apply to anchors as well. Because our focus is on self-localization, threats affecting anchors are manifested as either link threats (e.g., weak signal) or system threats (e.g., not enough threats). Note that the lists in the tables are not meant to be complete; the threat analysis depends on the specific use case. Furthermore, some threats can overlap or propagate. For example, software failure of an anchor could cause a weak signal (link), which in turn might result in a shortage of anchors (system). Moreover, application-specific threats can be implied by native threats (e.g., in a robot-assisted warehouse, one malfunctioning anchor could affect the accuracy of the localization service, which might lead to collisions between robots and humans).

## 4. Metrics and Trustworthiness Indication

**Figure 4:**
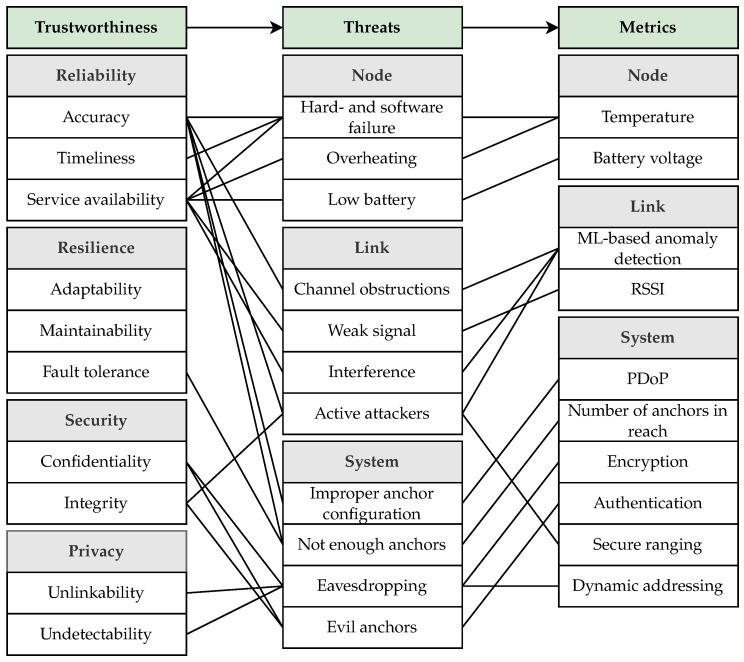
Mapping of trustworthiness attributes to metrics through threats.

Metrics are used to evaluate the characteristics of a system in terms of its respective attributes. As the attribute definition alone is too general to determine meaningful and relevant metrics, metrics that enable the detection of the previously introduced threats are chosen. The approach of mapping metrics to attributes for the discussed UWB self-localization system is depicted in Figure 4.

As each chosen metric has a different dynamic range, they are not comparable a priori; thus, mapping them to trustworthiness indicators with specific conditions is required. To further extract high-level evaluation (e.g., the trustworthiness with respect to reliability), such indicators can be unified into trustworthiness indices.

In this section, the general notation of metrics, their mapping to trustworthiness indicators, and their unification into trustworthiness indices is provided. In the following section, the metrics are discussed one by one.

### 4.1. Metrics

In this work, measurable quantities that are available from the UWB transceiver or from intermediate information within the localization scheme are selected. Those metrics, which are detailed in the subsequent section, are temperature (temp), battery voltage (bat), ML-based anomaly detection [12] (ml), RSSI (rssi), position dilution of precision (PDoP, pdop), number of anchors in reach (na), encryption used (enc), authentication used (auth), secure ranging used (sr), and dynamic addressing used (da). Metrics are denoted by $m_{i}$, where *i* is the abbreviation of the metric (e.g., $m_{\mathrm{rssi}}$ for RSSI), or simply by *m*.

For later use, the following sets are defined according to measurements that relate to the node (temp and bat), link with an anchor (ml and rssi), or system configuration (all remaining metrics).

$$\begin{array}{cccc} M_{\mathrm{node}} & =\left\{m_{\mathrm{temp}},m_{\mathrm{bat}}\right\} & \; & \mathrm{node}\;\mathrm{metrics}\\ M_{\mathrm{link}} & =\left\{m_{\mathrm{ml}},m_{\mathrm{rssi}}\right\} & \; & \mathrm{link}\;\mathrm{metrics}\\ M_{\mathrm{sys}} & =\left\{m_{\mathrm{pdop}},m_{\mathrm{na}},m_{\mathrm{enc}},m_{\mathrm{auth}},m_{\mathrm{sr}},m_{\mathrm{da}}\right\} & \; & \mathrm{system}\;\mathrm{metrics}\\ M & =M_{\mathrm{node}}\cup M_{\mathrm{link}}\cup M_{\mathrm{sys}} & \; & \mathrm{all}\;\mathrm{metrics} \end{array}$$

For $m\in M_{\mathrm{link}}$, $m^{\left(A\right)}$ denotes the metric measurement between the node and the anchor $A\in A_{\mathrm{eval}}$. Note that a metric can be either real valued m∈R (e.g., temperature readings), or binary with m∈{state1,state2} (e.g., encryption on/off).

### 4.2. Mapping to Trustworthiness Indicators

**Figure 5:**
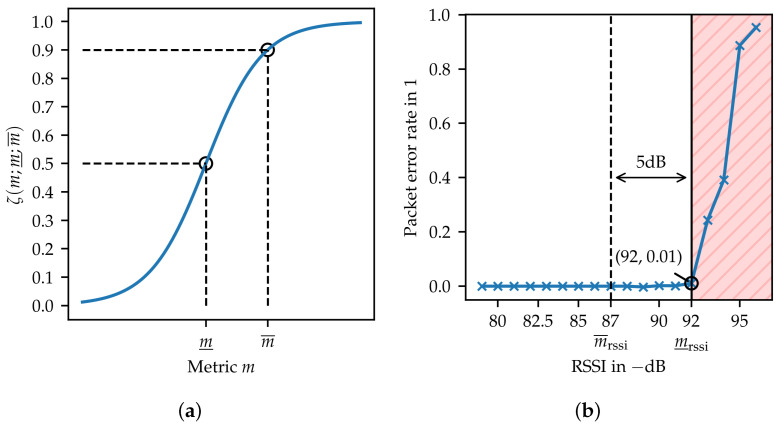
(**a**) The sigmoid function is used to map metrics to a trustworthiness indicator. (**b**) The RSSI is used to account for anchors with low signal strength that might not respond. The area considered not trustworthy is illustrated in red.

In this step, the selected metrics m∈M are mapped to unified trustworthiness indicators $T_{m}$ bound to the interval [0,1]. To simplify the notation of superscripts and subscripts, e.g., $T_{\mathrm{ml}}$ is written instead of $T_{m_{\mathrm{ml}}}$. Similarly, for A∈A, $T_{{\mathrm{ml}}^{\left(A\right)}}$ is used instead of $T_{m_{\mathrm{ml}}^{\left(A\right)}}$. This normalization is required to account for the individual sensitivity of the metrics and their application-specific importance. For real value metrics, a sigmoid function

(3) $$T_{m}=\zeta \left(m;\underline{m},\bar{m}\right)=\frac{1}{1+e^{-g\frac{1}{\bar{m}-\underline{m}}\left(m-\underline{m}\right)}}\;$$

with the constant g=ln9 is used for the mapping (c.f. Figure 5a). The sigmoid function enables the appropriate mapping ζ:R→[0,1], ensuring that any real-valued input is transformed into a value within the interval [0,1]. Furthermore, the sigmoid function provides a non-zero gradient even in saturated regions, i.e., where $T_{m}>0.9$ or $T_{m}<0.1$. This characteristic is beneficial when the proposed framework is used in the context of trustworthiness management to account for changes in trustworthiness over time. The sensitivity can be adjusted by tuning parameters, such that $\underline{m}$ marks the transition from not trustworthy to trustworthy at $T_{m}=0.5$ while $\bar{m}$ is set to a value representing a reasonable level of trust, i.e., $T_{m}=0.9$. Binary metrics are mapped to 1 if they are considered trustworthy or to 0 otherwise.

### 4.3. Trustworthiness Index

To obtain a unified trustworthiness index for each attribute (i.e., reliability, resilience, security, and privacy), the trustworthiness indicators must first be combined. Here, trustworthiness is defined by the least trusted component. Therefore, the minimum function is chosen as the evaluation criterion. For $m\in M_{\mathrm{link}}$,

(4) $$T_{m}^{*}=\min\left\{T_{m^{\left(A\right)}}\;|\;A\in A\right\}\;.$$

The trustworthiness index per attribute is provided by

$$\begin{array}{cc} I_{\mathrm{rel}} & =\min\{T_{\mathrm{temp}},T_{\mathrm{ml}}^{*},T_{\mathrm{pdop}},T_{\sec},T_{\mathrm{bat}},T_{\mathrm{rssi}}^{*},T_{\mathrm{na}}\},\\ I_{\mathrm{res}} & =\min\{T_{\mathrm{na}}\},\\ I_{\sec} & =\min\{T_{\mathrm{enc}},T_{\mathrm{da}},T_{\mathrm{aut}},T_{\sec},T_{\mathrm{ml}}^{*}\},\\ I_{\mathrm{priv}} & =\min\{T_{\mathrm{da}}\}. \end{array}$$

Note that the corresponding trustworthiness indicators are found through the relations in Figure 4. Reliability consists of accuracy, timeliness, and service availability. Accuracy links to the threats of hardware or software failure, channel obstruction, active attackers, improper anchor configuration, and not enough anchors. The corresponding metrics (and consequently indicators) are temperature, ML-based anomaly detection, PDoP, and number of anchors. The indicators for timeliness and service availability are found similarly. Most notably, the same metrics can be used for quantification of different attributes.

An overall trustworthiness index is provided by

$$I=\min\{I_{\mathrm{rel}},I_{\mathrm{res}},I_{\sec},I_{\mathrm{priv}}\}.$$

### 4.4. Trustworthiness-Enhanced Anchor Selection Scheme

Conventionally, self-localization is performed with all anchors in communication range to the node (here referred to as the *basic* approach). However, intermediate results of link level trustworthiness indicators (4) may be directly used to select only anchors that possess trustworthy links with the node (here referred to as the *sequential* approach). This distinction is formalized by the definition of $A_{\mathrm{eval}}$ (c.f. Section 2.1):

$$\begin{array}{c} A_{\mathrm{eval}}=\left\{\begin{array}{cc} A & \mathrm{if}\;\mathrm{the}\;\mathrm{basic}\;\mathrm{method}\;\mathrm{is}\;\mathrm{used},\\ \left\{A\in A\;|\;\min_{m\in M_{\mathrm{link}}}T_{m^{\left(A\right)}}\geq 0.5\right\} & \mathrm{if}\;\mathrm{the}\;\mathrm{sequential}\;\mathrm{method}\;\mathrm{is}\;\mathrm{used}. \end{array}\right. \end{array}$$

## 5. Selected Trustworthiness Indicators

The metrics that are linked to the threats, depicted in Figure 4, are introduced below. By inspecting the metric values, the thresholds required for mapping to the trustworthiness indicator in (3) are derived.

### 5.1. Temperature ($T_{\mathrm{temp}}$)

Temperature is used to detect overheating from external sources or to indicate hardware or software failures. The maximum operation temperature specified in the UWB transceiver datasheet is used as the mapping parameter ${\underline{m}}_{\mathrm{temp}}=$ = 85 °C. The second parameter is set to ${\bar{m}}_{\mathrm{temp}}={\underline{m}}_{\mathrm{temp}}-10\;{}^{\circ}C=75\;{}^{\circ}C$.

### 5.2. Battery Voltage ($T_{\mathrm{bat}}$)

**Figure 6:**
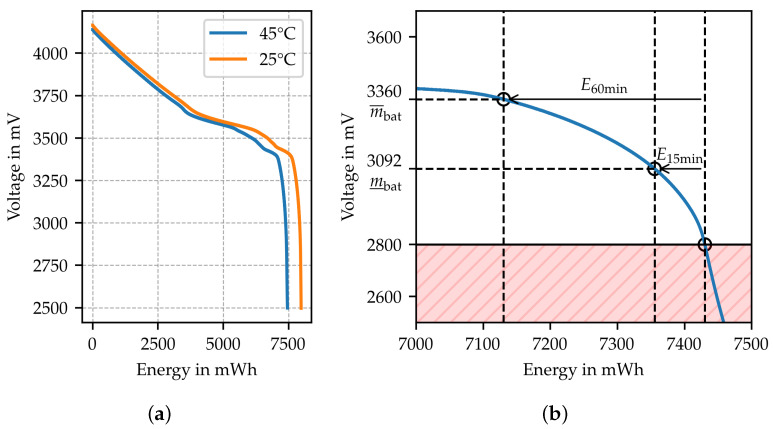
(**a**) Dischargecurve of an INR 18650-20R type battery [22] used to supply the node. (**b**) The discharge curve for 45 °C is used to derive tuning parameters for mapping the battery voltage to the corresponding trustworthiness indicator. The area deemed not trustworthy is illustrated in red.

Battery voltage is used as a metric to indicate the low battery threat and resulting issues with service availability. Through discharge curves of batteries captured for the typical node current (c.f. Figure 6a), the remaining battery time can be estimated [21]. Battery voltage measurements are required as input to conclude the current battery charge.

The minimum operation voltage of the node (2.8 V) can be used to find the tuning parameters ${\underline{m}}_{\mathrm{bat}}$ and ${\bar{m}}_{\mathrm{bat}}$. They are set according to allow further operation for time spans of 15 min and 60 min, respectively. This is achieved by first estimating the corresponding amounts of energy consumed by the node $E_{15\min}$ and $E_{60\min}$. In Figure 6b, from the intersection of the minimum operation voltage with the discharge curve, the curve is traced back by $E_{15\min}$ and $E_{60\min}$ to obtain the corresponding voltage levels used as tuning parameters, which are found to be ${\underline{m}}_{\mathrm{bat}}=3092\;mV$ and ${\bar{m}}_{\mathrm{bat}}=3360\;mV$. The discharge curve for 45 °C is used to account for the increased temperature with respect to the ambient temperature during operation.

### 5.3. ML-Based Anomaly Detection ($T_{\mathrm{ml}}$)

**Figure 7:**
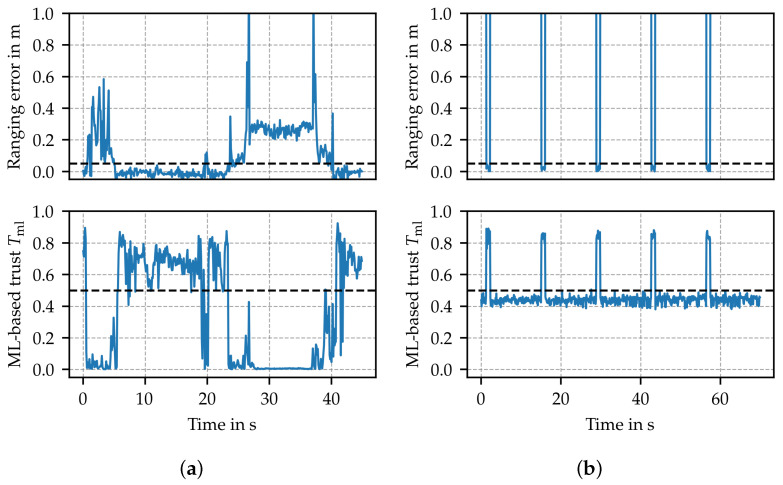
Performance of ML-based anomaly detection score in the case of (**a**) channel manipulation by a person temporarily blocking line of sight and (**b**) SHR attack active approximately 90% of time. The dashed black lines represent the thresholds used to differentiate between trustworthy and untrustworthy states.

ML-based anomaly detection facilitates channel impulse responses estimated at the receiver to effectively detect channel obstructions (Figure 7a), accounting for localization accuracy. The method evaluates the distance between key features of the CIR from known trustworthy channels to key features of the current channel realization. Thus, it indicates if the channel behaves as intended. Figure 7b reveals that ML-based anomaly detection can also detect spoofing attacks (e.g., SHR attack [18]), i.e., active attackers and interferers. The algorithm itself uses a sigmoid function at the output; hence, additional mapping is not required. The trustworthiness index is defined as

$$T_{\mathrm{ml}}=m_{\mathrm{ml}}=f_{\mathrm{ml}}\left({\hat{h}}_{a},{\hat{h}}_{b},{\hat{h}}_{c}\right),$$

with the autoencoder $f_{\mathrm{ml}}$ as defined, trained and validated in [18].

### 5.4. Received Signal Strength Indicator ($T_{\mathrm{rssi}}$)

To identify anchors that may not be responding reliably, affecting service availability, the RSSI reported by the UWB transceiver is assigned to $m_{\mathrm{rssi}}$. In Figure 5b, the packet error rate is plotted over the RSSI. The tuning parameter ${\underline{m}}_{\mathrm{rssi}}$ was chosen according to an acceptable packet error rate of 1%. The evaluation of a UWB dataset collected in an office environment [23] shows that the path loss caused by typical obstacles (i.e., people, bookshelves) does not exceed 5 dB. Thus, to ensure sufficient signal strength in varying indoor scenarios, ${\bar{m}}_{\mathrm{rssi}}={\underline{m}}_{\mathrm{rssi}}+5$ dB is used as trustworthy condition. Finally, the mapping parameters are ${\underline{m}}_{\mathrm{rssi}}=-92$ dB and ${\bar{m}}_{\mathrm{rssi}}=-87$ dB.

### 5.5. Position Dilution of Precision ($T_{\mathrm{pdop}}$)

PDoP provides an indication of the accuracy that can be achieved based on the placement of available anchors with respect to the estimated node position. It can be roughly interpreted as a ratio of position error to range error. Remember the anchors used for localization $A_{\mathrm{eval}}=\left\{A_{1},\ldots ,A_{K}\right\}$ and $A\in A_{\mathrm{eval}}$.

At first, the vectors

$$c^{\left(A\right)}=x^{\left(A\right)}-\hat{x},$$

pointing from the estimated node position $\hat{x}$, (2), to the respective anchor positions $x^{\left(A\right)}$ are defined. These vectors $c^{\left(A\right)}$ are then normalized and collected in the rows of matrix

$$D={\left[\begin{array}{c} \frac{c^{\left(A_{1}\right)}}{∥c^{\left(A_{1}\right)}∥}\;┆\;\cdots \;┆\;\frac{c^{\left(A_{K}\right)}}{∥c^{\left(A_{K}\right)}∥} \end{array}\right]}^{⊤}.$$

**Figure 8:**
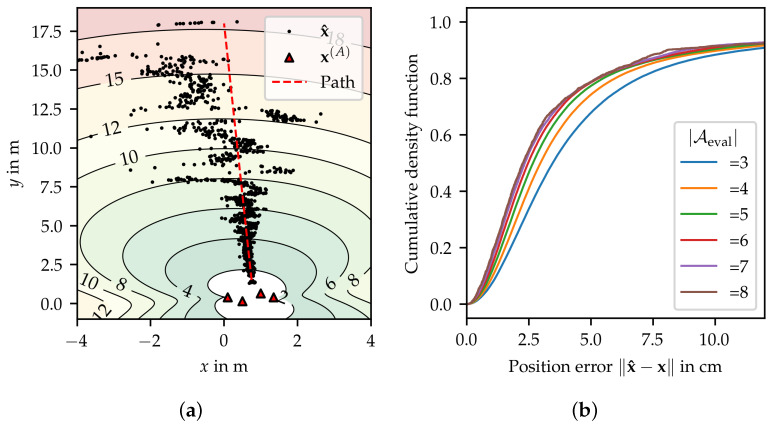
(**a**) Localization scenario with improper anchor configuration. The map shows levels of PDoP in the range from 2 to 18. (**b**) Cumulative density function of position error with respect to the number of anchors used.

Finally, according to [24], the metric

$$m_{\mathrm{pdop}}=\sqrt{\mathrm{trace}\left({\left(D^{⊤}D\right)}^{-1}\right)}$$

is defined. Table 4 reveals that good performance of the localization system can be expected for $m_{\mathrm{pdop}}<3$, while moderate performance can be achieved up to $m_{\mathrm{pdop}}<10$. Figure 8a shows position estimates $\hat{x}$ of a node moving along a linear path collected in an experiment. Based on the experiment, scattering of position estimates significantly increases for $m_{\mathrm{pdop}}>8$. The mapping was defined as

$$T_{\mathrm{pdop}}=\left\{\begin{array}{cc} \zeta \left(m_{\mathrm{pdop}},{\underline{m}}_{\mathrm{pdop}},{\bar{m}}_{\mathrm{pdop}}\right) & \mathrm{if}\;|A_{\mathrm{eval}}|\geq 3,\\ 1 & \mathrm{otherwise}. \end{array}\right.$$

with tuning parameters ${\underline{m}}_{\mathrm{pdop}}=8$ and ${\bar{m}}_{\mathrm{pdop}}=3$.

### 5.6. Number of Anchors ($T_{\mathrm{na}}$)

The number of anchors in reach

$$m_{na}=\left|A_{\mathrm{eval}}\right|$$

provides a measure of redundancy. At least three distance estimates, i.e., available anchors, are required for 2D localization. Having more anchors available increases the service’s fault tolerance. Figure 8b shows that a higher number of anchors can also improve the accuracy to a limited extent. The mapping was chosen with ${\underline{m}}_{\mathrm{na}}=3$ and ${\bar{m}}_{\mathrm{na}}=4.5$.

### 5.7. Binary Trustworthiness Indicators ($T_{\mathrm{auth}}$, $T_{\mathrm{enc}}$, $T_{\mathrm{sr}}$ and $T_{\mathrm{da}}$)

Binary metrics measure the system state by checking whether authentication, encryption, secure ranging, or dynamic addressing is used. Authentication and encryption are implemented using authenticated encryption with associated data according to the IEEE802.15.4 standard, ensuring data integrity and confidentiality. Secure ranging, an enhancement using the STS option from the IEEE802.15.4z standard, partially protects against several physical layer attacks. Dynamic addressing involves nodes and anchors changing their identifiers pseudo-randomly after each ranging cycle, increasing privacy by obfuscating the identities of communicating devices. As the use of each scheme adds extra protection, the mapping to the binary trustworthiness indicator $T^{\prime }$ is

$$T^{\prime }=\left\{\begin{array}{cc} 1 & \mathrm{if}\;\mathrm{the}\;\mathrm{corresponding}\;\mathrm{scheme}\;\mathrm{is}\;\mathrm{used},\\ 0 & \mathrm{otherwise}. \end{array}\right.$$

Note that it is imperative to evaluate them continuously, even if these indicators reflect system settings, as sophisticated attacks may alter these settings.

## 6. Evaluation

**Figure 9:**
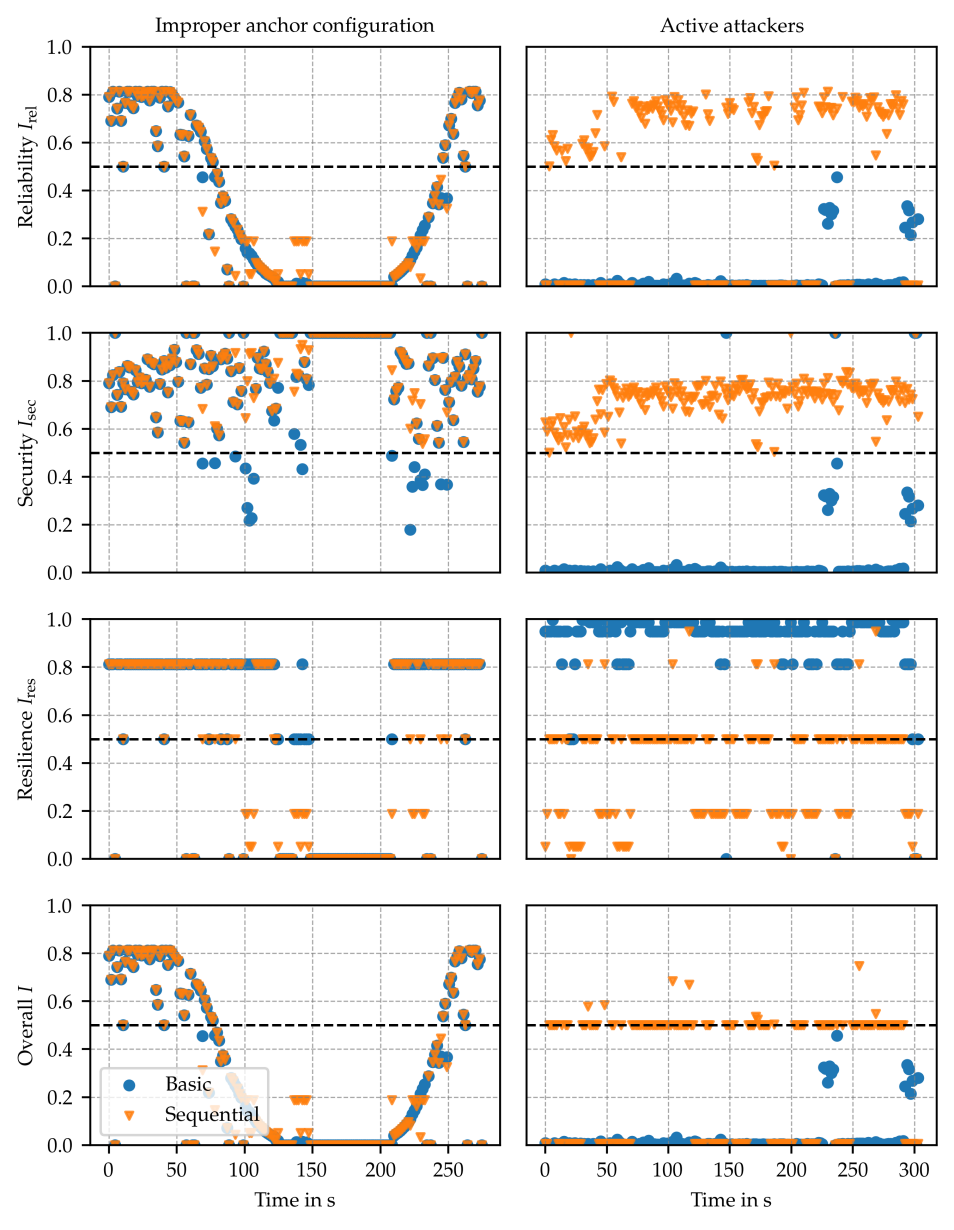
Trustworthiness evaluation on two threats. For the threat of improper anchor configuration, the distance from the node to a set of anchors is increased (**left**). For the threat of active attackers, four out of eight anchors were subjected to SHR jamming (**right**).

**Figure 10:**
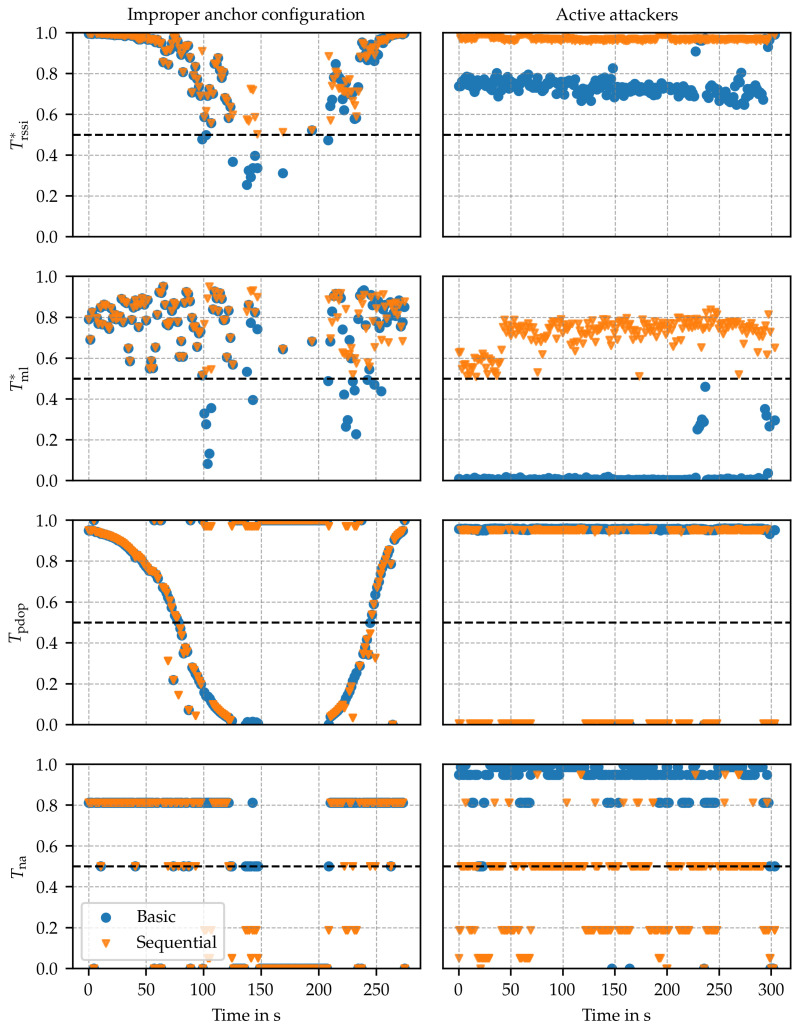
Trustworthiness indicators during evaluation of improper anchor configuration scenario (**left**) and active attackers scenario (**right**). For depiction, a subset of indicators is selected for which significant changes can be observed. These are the combined link indicators $T_{\mathrm{rssi}}^{*}$ and ML-based anomaly detection $T_{\mathrm{ml}}^{*}$ as well as the system indicators of PDoP $T_{\mathrm{pdop}}$ and number of anchors $T_{\mathrm{na}}$.

The trustworthiness assessment is designed to reflect the system’s operational trustworthiness across various attributes, including reliability, security, privacy, resilience, and safety. This assessment is not tailored to address specific threats; instead, a threat analysis similar to sensitivity analysis is conducted to identify relevant parameters. Ideally, this process encompasses all significant observation parameters that indicate proper system behavior. Consequently, the trustworthiness assessment must be capable of signaling low trustworthiness in the presence of any threat identified in Section 3 as well as detecting unknown system issues that impact these parameters. In this evaluation, the influence of two specific threats on the trustworthiness assessment is demonstrated, namely, improper anchor configuration and active attackers (c.f. Section 3). One and the same implementation of the trustworthiness framework was used for both scenarios without any tuning to the specific conditions of each scenario. The machine learning-based anomaly detection indicator was trained using a dataset [23] comprising range estimates captured in line-of-sight conditions across different environments (an auditorium and a private workshop) than those used in this work. The results are summarized in Figure 9 and Figure 10, where trustworthiness indices ($I_{\mathrm{rel}},I_{\sec},I_{\mathrm{res}}$ and *I*) and trustworthiness indicators ($T_{\mathrm{rssi}}^{*},T_{\mathrm{ml}}^{*},T_{\mathrm{pdop}}$ and $T_{\mathrm{na}}$) that are significant for the selected scenarios are depicted. In both scenarios, the assessment methods (c.f. Section 4.4) basic (colored in blue) and sequential (colored in orange) are compared against each other.

### 6.1. Improper Anchor Configuration

In this experiment, corresponding to the setup used in Figure 8a, the node first approaches the borders of the service area covered by a set of four anchors, exceeds them, and then returns to its starting position at a maximum distance of approximately 20 m from the anchors. Initially, the anchors are favorably located, i.e., ($T_{\mathrm{pdop}}$) is low, and the signal strengths ($T_{\mathrm{rssi}}^{*}$) are high, resulting in a high reliability index ($I_{\mathrm{rel}}$) dominated by the number of anchors available ($T_{\mathrm{na}}$). As the distance to the anchors increases, the dilution of precision $T_{\mathrm{pdop}}$ and signal strength $T_{\mathrm{rssi}}^{*}$ decrease. After 50 s, $T_{\mathrm{pdop}}$ starts to dominate the reliability index, causing $I_{\mathrm{rel}}$ to decline. This decline in trustworthiness corresponds to a loss in accuracy due to the dilution of precision effect. After approximately 75 s, the index reaches the threshold, signaling the transition to an untrustworthy state. As the node moves further away from the anchors, the signal strength $T_{\mathrm{rssi}}^{*}$ after 100 s also reaches the threshold, i.e., when reduced service availability is anticipated. Breaking links reduce the number of anchors $T_{\mathrm{na}}$ and cause the system to fail.

With this experiment, the framework’s ability to classify the system’s state as untrustworthy well before a complete loss of localization service occurs is demonstrated. This early detection provides an opportunity to implement countermeasures in a timely manner. Recall that the sequential assessment method differs from the basic method by selecting the subset of anchors with trustworthy link indicators (c.f. Section 4.4). In this experiment, both assessment methods performed similarly. This is due to the fact that $T_{{\mathrm{ml}}^{\left(A\right)}}$ for all A∈A stayed at a high level as well as $T_{{\mathrm{rssi}}^{\left(A\right)}}$ for all A∈A were at similar levels at any point in time.

### 6.2. Active Attackers

In this scenario, eight anchors are used in a static office environment, positioned at distances ranging from 1.5 m to 4 m to the node. A jamming device in proximity of the node is executing an SHR attack on four out of eight anchors, aiming at the reduction of estimated distances, which further yields wrong position estimates. Using the basic method, $I_{\mathrm{rel}}$ and $I_{\sec}$ are both low due to low values $T_{\mathrm{ml}}^{*}$ for anchors being subject to jamming. However, $I_{\mathrm{res}}$ is high due to a larger anchor set $A_{\mathrm{eval}}$, also reflected in $T_{\mathrm{na}}$.

Using the sequential method, in the first step, the trustworthiness of anchor links is evaluated. Based on the subset of trustworthy anchors, in the second step, the location estimate, the remaining trustworthiness indicators and indices are computed. In $I_{\sec}$, this is reflected by maintaining trustworthiness at a level of approximately 0.6, originating from $T_{\mathrm{ml}}$ of trusted anchors, i.e., from anchors not subject to jamming. However, by considering a lower number of anchors for the evaluation, $I_{\mathrm{res}}$ is around 0.5, and for some measurements, it is even lower. Despite the lower resilience value, the overall trustworthiness index *I* indicates that the sequential method can maintain tolerable trustworthiness during the attack on 4 out of 8 anchors.

SensorsThis benefit can also be seen in the accuracy. While the root mean square error in the attack scenario would result in approx. 81 cm, the basic method classifies all estimates as untrustworthy. The sequential method achieves a root mean square error of 17 cm with a trustworthiness index I≥0.5. In only 39% of the estimates, it obtains I<0.5.

### 6.3. Findings

This subsection summarizes the essential findings from the experimental evaluation.

- In both examples, $I_{\mathrm{rel}}$ and $I_{\sec}$ precisely detect threats to availability, accuracy, and integrity. Because reliability and security are attributes that support safety, this also indicates that the proposed method can detect certain safety threats.
- The improved reliability $I_{\mathrm{rel}}$ and security $I_{\sec}$ of the *sequential* method in the active attacker use case underlines the potential of using intermediate results from the trustworthiness assessment to increase service performance.
- In order to provide a holistic prediction of the system’s proper operation, trustworthiness assessment through carefully selected metrics is essential. Several of these metrics influence multiple attributes, resulting in an interconnected evaluation that advances beyond the isolated analysis of each attribute.
- From changes within the trustworthiness indices, it is possible to predict system vulnerabilities before the actual occurrence of failures; c.f. $I_{\mathrm{rel}}$ in Section 6.1. Hence, trustworthiness has a high potential to be leveraged as an early warning mechanism. Furthermore, this early warning offers the possibility of taking countermeasures to maintain the level of trustworthiness in the system.

## 7. Conclusions

In this work, a method that systematically links the general definition of trustworthiness to an evidence-based trustworthiness index is proposed. The focus is on UWB self-localization, a critical service in the IoT domain. The threat-driven metric selection approach represents the first holistic assessment of trustworthiness concerning reliability, security, privacy, and resilience. While safety is often seen as an additional attribute, in the context of UWB self-localization its characteristics are considered to be supported by reliability and security.

The proposed method, which connects trustworthiness definitions and attributes to threats, metrics, and trustworthiness indicators and indices, has the potential to serve as a general framework. While future work may extend the threat analysis and metric selection, the proposed approach demonstrates the functional principle. Interestingly, the interconnection of attributes through individual metrics indicates that a holistic evaluation of trustworthiness surpasses the isolated analysis of individual attributes.

Experimental analysis shows that using intermediate trustworthiness indicators can improve service quality. Comparing conventional UWB self-localization with an enhanced method that applies a trustworthiness-based anchor selection scheme reveals clear localization performance improvements without additional costs. Furthermore, while many traditional metrics are model-based or derived from information theory, ML techniques can also significantly contribute to the assessment of trustworthiness.

In conclusion, the presented method proposes a systematic approach for holistic trustworthiness assessment. By leveraging intermediate results and incorporating advanced techniques such as ML-based metrics, substantial improvements in system performance can be achieved, highlighting the potential for future advancements.

## Figures and Tables

**Table 1:** Overview of node threats. Every second row is colored grey to improve readability.

Threats	Examples	Impact
Hardware and software failures	Software crashes, invalid configuration, firmware corruption, physical destruction, harsh environmental conditions	Node downtime, node malfunction, additional maintenance costs
Overheating	Misconfigurations, bugs, poor ventilation	Decreased ranging accuracy, higher power consumption, fire hazard, shorter lifespan of the device
Low battery	Incorrect power consumption settings, environmental conditions, battery aging, mismanagement of recharging	Decreased anchor performance, anchor downtime

**Table 2:** Overview of link threats. Every second row is colored grey to improve readability.

Threats	Examples	Impact
Channel obstructions	Reflections, non line-of-sight	Decreased ranging accuracy
Weak signal	Large distance between an anchor and a node	Higher packet error rate, low data throughput
Interference	Unintentional interference (in-band or out-band), jamming	Decreased ranging accuracy, denial of service, increased error rates
Active attackers	Jamming, packet injection, preamble tampering, active probing, denial of service, payload overwriting	Compromised security and reliability

**Table 3:** Overview of system threats. Every second row is colored grey to improve readability.

Threats	Examples	Impact
Improper anchor configuration	Incorrect anchor placement	Decreased localization accuracy, degraded system performance
Not enough anchors	Insufficient number of anchors for unambiguous localization	Localization service fails
Eavesdropping	A passive attacker with a UWB receiver	Compromised confidentiality, privacy breaches of localization data
Evil anchors	Impersonating anchors and announcing wrong time information or wrong anchor position	Compromised security and reliability

**Table 4:** Rating of PDoP values [24].

$m_{\mathrm{pdop}}$	<1	1–2	2–5	5–10	10–20	>20
Rating	Ideal	Excellent	Good	Moderate	Fair	Poor

## Data Availability

The data used in the evaluation section was published in [25].
